# Delayed diagnosis resulting in increased disease burden in multiple myeloma: the legacy of the COVID-19 pandemic

**DOI:** 10.1038/s41408-023-00795-w

**Published:** 2023-03-15

**Authors:** Jonathan Carmichael, Frances Seymour, Graham McIlroy, Sarrah Tayabali, Rosie Amerikanou, Sylvia Feyler, Rakesh Popat, Guy Pratt, Christopher Parrish, A. John Ashcroft, Graham H. Jackson, Gordon Cook

**Affiliations:** 1 Leeds Institute of Clinical Trial research & Leeds Cancer Centre, University of Leeds, Leeds, UK; 2 NIHR Medtech & In Vitro Diagnostics Cooperative (Leeds), Leeds, UK; 3 Dept of Haematology, Leeds Cancer Centre, Leeds Teaching Hospitals Trust, Leeds, UK; 4 University Hospitals Birmingham NHS Foundation Trust, Birmingham, UK; 5 Dept of Haematology, University College London Hospitals NHS Foundation Trust, London, UK; 6 Dept of Haematology, Calderdale & Huddersfield Foundation Trust, Huddersfield, UK; 7 Dept of Haematology, Pinderfields Hospital, Mid Yorkshire NHS Trust, Wakefield, UK; 8 Department of Haematology, Newcastle University, Newcastle, UK; 9 College of Myeloma (UK), London, UK

**Keywords:** Diagnosis, Signs and symptoms

## Abstract

The COVID-19 pandemic has had global healthcare impacts, including high mortality from SARS-CoV-2 infection in cancer patients; individuals with multiple myeloma (MM) are especially susceptible to poor outcomes. However, even for MM patients who avoided severe infection, the ramifications of the pandemic have been considerable. The consequences of necessary socio-geographical behavior adaptation, including prolonged shielding and interruptions in delivery of non-pandemic medical services are yet to be fully understood. Using a real-world dataset of 323 consecutive newly diagnosed MM patients in England, we investigated the impact of the COVID-19 pandemic on routes to myeloma diagnosis, disease stage at presentation and relevant clinical outcomes. We demonstrate increasing MM presentations via emergency services and increased rates of bony and extra-medullary disease. Differences were seen in choice of induction therapy and the proportion of eligible patients undertaking autologous stem cell transplantation. Whilst survival was statistically inferior for emergency presentations, significant survival differences have yet to be demonstrated for the entire cohort diagnosed during the pandemic, making extended follow-up critical in this group. This dataset highlights wide-ranging issues facing MM patients consequent of the COVID-19 pandemic, with full impacts for clinicians and policy-makers yet to be elucidated.

## Introduction

Multiple myeloma often presents with symptoms both vague and non-specific, creating a diagnostic challenge. Consequently, the diagnosis of myeloma is frequently made via emergency services and may follow multiple prior consultations with medical professionals. The National Cancer Intelligence Network (NCIN) “Routes to Diagnosis” report identified that 32% of myeloma diagnoses in 2013 were made via emergency care, with these emergency presentations being associated with shorter survival [1–3]. Furthermore, patients presenting via the emergency route are likely to exhibit more CRAB features or higher International Staging System (ISS) scores, indicating a higher burden of disease [4]. Patient-reported accounts indicate between 1 and 10 primary care consultations prior to diagnosis with a period of 2-17 months between first seeking help for relevant symptoms and the diagnosis of MM being made [5], while a retrospective case review reported a delay in diagnosis was associated with detrimental impact on clinical course and outcome [6].

Escalating transmission rates of severe acute respiratory syndrome coronavirus 2 (SARS-CoV-2) causing Coronavirus disease 2019 (COVID-19) rapidly resulted in the evolution of a global pandemic, leading to 48,539,872 known infections, and 1,232,791 deaths in 215 countries by the end of 2020 [7]. Infection with SARS-CoV-2 has been characterized by differential outcomes, influenced in part by age, sex, ethnicity and co-morbidity profiles. Early in the pandemic, studies suggested that cancer patients, especially blood cancer and myeloma specifically, were more likely to experience severe clinical sequelae from infection [8–10]. As a consequence, altered diagnostic and treatment pathways were developed for MM patients, intended to minimize potential exposure to SARS-CoV-2. However, relative to the existing pathways, these may have inadvertently further disadvantaged a population of patients already disproportionately impacted by the influence of the pandemic on routine care, particularly in terms of delayed, amended or abandoned GP and hospital appointments, diagnostic procedures and in-hospital therapies {8-9].

During the early months of the COVID-19 pandemic in the UK, a 70% fall in urgent referrals for suspected cancer occurred [11] and 24% fewer patients contacted primary care with possible cancer symptoms [12]. It is estimated that 430,000 fewer patients were referred on suspected cancer pathways between March 2020 and February 2020 compared to the previous year (https://www.cancerresearchuk.org/health-professional/diagnosis/hp-covid-19-and-cancer-hub#HP_COVID-190). Furthermore, the pandemic has led to a fundamental and permanent shift in methods of delivery of primary care, with an increased focus on symptom screening and telemedicine that may result in challenges in the assessment of patients with vague symptoms. As such, the full impact of the COVID-19 pandemic on cancer care and outcomes will take many years to become apparent. Given the known detrimental impact of a myeloma diagnosis occurring via an emergency route and the difficulties the non-specific nature of myeloma symptoms pose to clinical assessment, we investigated the impact of the COVID-19 pandemic on the route to myeloma diagnosis, disease stage at presentation and patient outcomes.

## Materials & methods

### Design and Participants

We conducted a fully anonymised, clinical audit of consecutive newly diagnosed patients with MM from January 2019 until July 2021 across 5 institutions (3 University and 2 large District hospitals) in England, UK covering a population of approximately 10 million. Full details of presentation, disease and patient-related parameters were obtained from hospital electronic patient records (EPR). These included extent of disease, route to diagnosis, anti-myeloma therapy and initial response to therapy. Timing of first myeloma symptom was extracted from hospital clinic letters, rather than from GP records. Extracted data were collated centrally at the University of Leeds where statistical analysis was performed. The audit was carried out in accordance with local guidelines and in compliance with the principles of the Declaration of Helsinki and the Good Clinical Practice guidelines of the International Conference on Harmonization.

### Myeloma characterization

Myeloma was classified as symptomatic or smoldering according to the revised IMWG criteria [13]. Genetic risk was assessed according to UK guidelines [14]. High risk cytogenetics were considered to be t(4:14), t(14:16), t(14:20), deletion of 17p, deletion of 1p and gain/amp of 1q. Frailty was determined in accordance with the IMWG Frailty Score (FS) and the UK Myeloma Research Alliance Myeloma risk profile (UKMRA MRP) was calculated as described previously [15, 16].

### Statistical analysis

Data were summarized with descriptive statistics and no imputation was performed in the event of missing data. Continuous variables were reported as medians and interquartile ranges (IQR) and categorical variables reported as a percentage and absolute number of patients. Fisher’s exact test was used to compare outcomes between categorical variables, Mann-Whitney-U test for ordinal variables, t-tests for continuous variables and log-rank for survival data. Two-sided *P* < 0.05 was considered significant.

## Results

### Patient characteristics

From January 2019, 323 consecutive newly diagnosed MM patients were identified. Patient characteristics are presented in Table 1. Patients were cohorted in accordance with the date when the 1st UK COVID-19 index case was reported (31st of January 2020). Consequentially, consecutive patients diagnosed between 1st January 2019 until 31st January 2020 were described as the pre-COVID cohort (*n* = 110) and consecutive patients diagnosed from 1st February 2020 until 31st July 2021 were described as the post-COVID cohort (*n* = 213). The median age of the complete cohort was 71 years (range 28–93) with 24% 80 years or older. No significant difference in age at presentation was evident between cohorts (Table 1). There was no significant difference in the male:female ratio between the cohorts (pre-COVID ratio 1.56 compared with post-COVID ratio 1.45).

**Table 1:** Baseline patient characteristics of all (*n* = 323) those diagnosed from 01 January 2019–31 January 2020 (Pre-COVID, *n* = 110) and those diagnosed from 01 February 2020–31 July 2021 (Post-COVID, *n* = 213).

		All (*n* = 323)	Pre-COVID (*n* = 107)	Post-COVID (*n* = 216)
Median age (IQR)		71 (62–79)	71 (61–78)	71 (62–80)
	<70	146 (45.2)	53 (48.2)	93 (43.7)
Age groups *N* (%)	70–79	98 (30.3)	33 (30.0)	65 (30.5)
	≥80	79 (24.5)	24 (21.8)	55 (25.8)
Sex: *N* (%) Male		193 (59.8)	67 (61.9)	126 (59.2)
Female		130 (40.3)	43 (39.1)	87 (40.9)
WHO PS: *N* (%)				
0		102 (31.6)	49 (44.6)	53 (24.9)
1		122 (37.8)	35 (31.8)	87 (40.9)
≥2		96 (29.7)	26 (23.6)	70 (32.9)
Missing		3 (0.9)	0 (0)	3 (1.4)
ISS stage: *N* (%)				
Stage I		103 (31.9)	39 (35.5)	64 (30.0)
Stage II		84 (26.0)	27 (24.5)	57 (26.8)
Stage III		114 (35.3)	38 (34.5)	76 (35.7)
Missing		32 (6.8)	6 (5.5)	16 (7.5)
Cytogenetic risk groups: *N* (%)				
Standard		58 (18.0)	5 (4.55)	53(24.9)
High risk		63 (19.5)	23 (20.9)	40 (18.8)
Missing		202 (62.5)	82 (74.6)	120 (56.3)
Isotype: *N* (%)				
IgG		187(57.9)	70(63.6)	117 (54.9)
IgA		60 (18.6)	16 (14.6)	44 (20.7)
LC		71 (22)	24 (21.8)	47 (22.1)
Non-secretory		3 (0.9)	0	3 (1.4)
Other		2 (0.6)	0	2 (0.9)
Ethnicity: *N* (%)				
White		248 (76.8)	167 (78.4)	81 (73.6)
Black		28 (8.7)	15 (7.0)	13 (11.8)
Asian		15 (4.6)	13 (6.1)	2 (1.8)
Other		10 (3.1)	6 (2.8)	4 (3.6)
Missing		22 (6.8)	12 (5.6)	10 (9.1)
mIMWG FS: *N* (%)				
Non-frail		51 (15.8)	24 (21.8)	27 (12.7)
Intermediate frailty		70 (21.7)	27 (24.6)	43 (20.2)
Frail		200 (61.9)	59 (53.6)	141 (66.2)
N/A		2 (0.6)	0 (0.0)	2 (0.9)
UKMRA MRP: *N* (%)				
Low Risk		110 (34.1)	39 (35.5)	71(33.3)
Intermediate risk		41 (12.7)	10 (9.1)	31(14.6)
High risk		125 (38.7)	35 (31.8)	90(42.3)
N/A		47 (14.6)	26(23.6)	21(9.9)
Symptomatic MM: *N (%)*				
Smoldering MM		58 (18.0)	17 (15.5)	41 (19.2)
Symptomatic MM		265 (82.0)	93 (84.5)	172 (80.8)

WHO PS: World Health Organization Performance Status. Standard risk was defined as the absence of any of the risk lesions; t(4;14), t(14;26), t(14;20), del(17p), del(1p) and gain(1q), high-risk was defined as one or more lesion present. High-risk and ultra-high-risk were combined and defined as high-risk for the analysis.

### Disease characteristics

For all patients, 58 (18%) were diagnosed as having smoldering multiple myeloma (sMM) with 265 (82%) diagnosed as MM requiring treatment, as per internationally agreed criteria [13]. There was no difference seen between the two cohorts (sMM: pre-COVID 17 (15.5%), post-COVID 41 (19.2%, *p* = 0.25). The level of availability of genetic risk assessment was low across the cohort, reflecting real-world practice but for patients who were assessed, prevalence of high-risk disease was similar between cohorts. During the pandemic, genetic testing did not cease in centers where it was routinely offered. A lower proportion of missing results was noted in the post-COVID cohort by comparison to pre-COVID cohort, which is likely to reflect the national roll-out of genetic testing outside of clinical trials in the U.K (Table 1). The most frequent genetic abnormalities were in chromosome 1 (Ch1p^loss^, Ch1q^gain^ and Ch1q^Amp^), present in 62.3% of defined genetically high-risk disease. Paraprotein isotypes were similar between the 2 cohorts with 22% of patients being diagnosed with light chain only MM (LCMM).

### Disease presentation

**Fig. 1:**
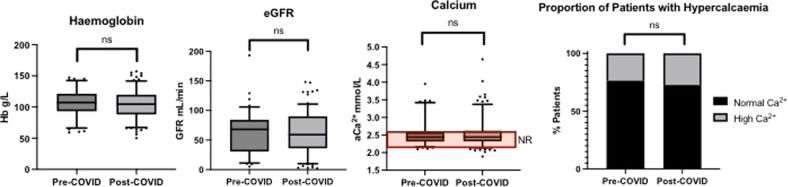
Presenting pathological features of symptomatic, newly-diagnosed myeloma patients. Those diagnosed between 1st January 2019–31st January 2020 (Pre-COVID, *n* = 93) and those diagnosed from 1st February 2020–31st July 2021 (Post-COVID, *n* = 172). Data presented as box plots of 95% confidence intervals. * *p* < 0.05, ns non-significant, NR normal range.

The clinical and pathological features at diagnosis were compared between the two cohorts. The number of patients presenting with symptoms attributable to MM was similar between cohorts (pre-COVID 79 (75.2%), post-COVID 164 (77.0%), *p* = 0.34). In patients with symptoms attributable to their myeloma, the median time from first symptoms to diagnosis was 3 months (range 0–26 months), with no difference noted between the cohorts (*p* = 0.86). The median presenting hemoglobin was 110 g/l (IQR 94–124) with no significant difference in presenting hemoglobin levels between groups (pre-COVID median 112 g/l (IQR 95-127), post-COVID median 108 g/l (IQR 92–123); *p* = 0.13; Fig. 1). The median presenting glomerular filtration rate (GFR) was 68 mls/min (range 2, 119)—similar across cohorts (pre-COVID median 70mls/min (IQR 39.25–84), post-COVID median 67mls/min (IQR 41.25–90; *p* = 0.84; Fig. 1)). The median presenting serum calcium concentration was 2.46 mmols/l (IQR 2.31–2.54) with no difference between cohorts (pre-COVID median 2.43 mmols/l (IQR 2.33–2.54-), post-COVID median 2.41 mmols/l (IQR 2.30–2.56); *p* = 0.97; Fig. 1) and similar rates of patients presenting with hypercalcaemia (pre-COVID 22 (20%), post-COVID 48 (22.5%); Fig. 1).

**Fig. 2:**
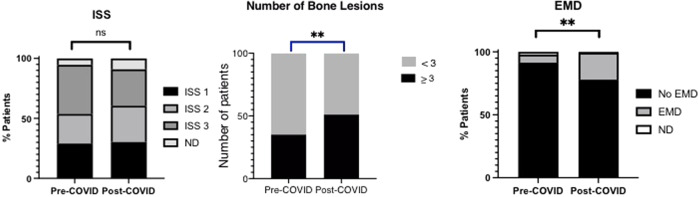
Presenting disease burden of newly diagnosed myeloma patients, those diagnosed from 1st January 2019–31st January 2020 (Pre-C, *n* = 110) and those diagnosed from 1st February 2020–31st July 2021 (Post-C, *n* = 213). *p < 0.05, **p < 0.01, ns Not Significant, ISS International Staging System, MBD myeloma bone disease (>3 lytic lesions) EMD extra-medullary disease.

**Fig. 3:**
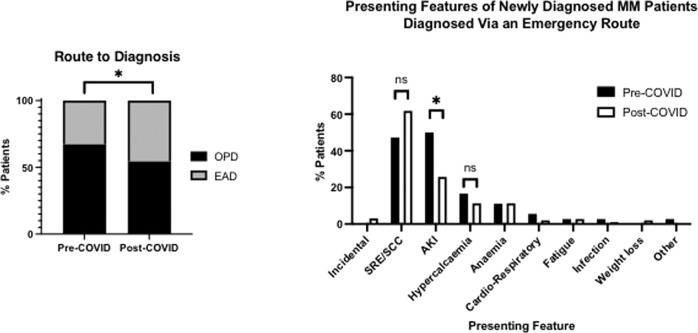
Route to diagnosis of newly diagnosed myeloma patients, those diagnosed from 1st January 2019–31st January 2020 (Pre-COVID, *n* = 110) and those diagnosed from 1st February 2020–31st July 2021 (Post-COVID, *n* = 213). *p < 0.05. OPD diagnosed through an outpatient consultation, EAD diagnosed through an emergency admission. SRE skeletal-related event, AKI acute kidney injury, SCC spinal cord compression, Cardio-Respiratory cardiovascular and/or respiratory compromise.

There was no identified difference in ISS stage between cohorts (*p* = 0.50). However, patients diagnosed after the UK COVID-19 index case (post-COVID cohort) demonstrated a significantly higher level of myeloma lytic bone disease, defined as ≥3 lytic lesions on cross-sectional imaging (pre-COVID 39 (35.5%), post-COVID 106 (49.7%); *p* < 0.01; Fig. 2) and a significantly higher incidence of extra-medullary disease (pre-COVID 6 (5.5%), post-COVID 37 (17.4%), *p* = 0.003, Fig. 2). For patients who were diagnosed after the onset of the COVID-19 pandemic, there was a significantly higher level of diagnosis through the emergency route compared to those diagnosed pre-pandemic (pre-COVID 36 (32.7%), post-COVID 97 (45.5%), *p* = 0.03, Fig. 3). The pattern of clinical complications precipitating emergency admission that resulted in the diagnosis had also changed between the two cohorts, with acute kidney injury accounting for the majority of emergency admissions in the pre-COVID cohort (*p* < 0.001) compared with skeletal-related events, including spinal cord compression, the predominant cause for diagnosis through the emergency route in the post-COVID cohort (Fig. 3).

### Patient fitness

**Fig. 4:**
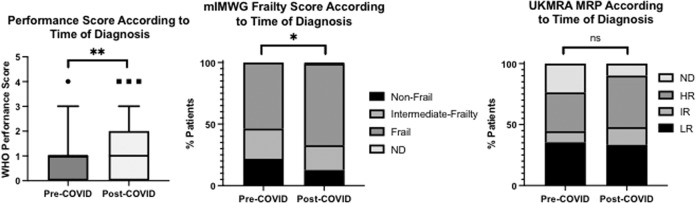
Patient fitness at diagnosis of MM, as defined by WHO performance score, mIMWG FS and UKMRA MRP, in those diagnosed from 1st January 2019–31st January 2020 (Pre-COVID, *n* = 110) and from 1st February 2020–31st July 2021 (Post-COVID, *n* = 213). HR high risk, IR intermediate risk, LR low risk *p < 0.05, **p < 0.005, ND no data.

Patient fitness at diagnosis was determined by a combination of WHO performance status (PS) and the presence of comorbidities, with the median PS of 1 (range 0–4) and median number of comorbidities of 2 (range 0–9). No significant difference between those diagnosed with smoldering MM (*n* = 58) and symptomatic MM (*n* = 265) was evident in either PS or comorbidities. In symptomatic MM, post-COVID patients had a significantly poorer PS compared to those diagnosed pre-pandemic (*p* = 0.002; Fig. 4). Using the modified IMWG FS, a larger proportion of those diagnosed after the onset of the pandemic had a higher frailty designation compared to those diagnosed pre-pandemic (pre-COVID 59 (54%) compared with post-COVID 141 (67%); *p* < 0.05, Fig. 4). The UKMRA MRP, developed and validated in clinical trial populations and validated in real-world populations [15, 17, 18] was applied to the current dataset: MRP mean scores were able to discriminate between IMWG frail patients (MRP mean score 0.036 (95%CI −0.009, 0.08); *p* < 0.0001) and IMWG non-frail patients (MRP mean: −0.534 (95%CI −0.59, −0.48; Supplementary Fig. 1). There was no identified statistically significant relationship between MRP score and pre- or post-COVID cohorts (*p* = 0.24).

### Outcomes

**Fig. 5:**
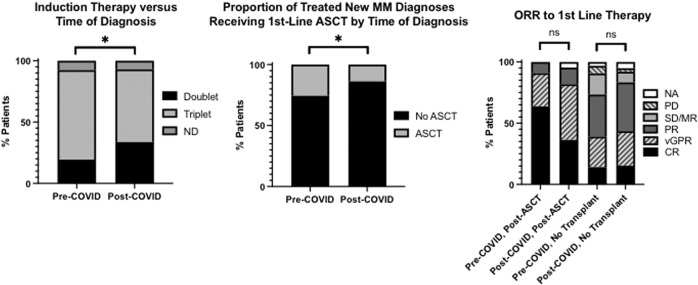
Induction treatment choice and ASCT at first line in those diagnosed from 1st January 2019–31st January 2020 (Pre-COVID, *n* = 86) and from 1st February 2020–31st July 2021 (Post-COVID, *n* = 160) and treated for MM. Overall response (ORR) to first line therapy, delineated by transplant eligibility and diagnosis cohort. ASCT autologous stem cell transplant, TE transplant eligible, TNE transplant ineligible, *p < 0.05.

**Fig. 6:**
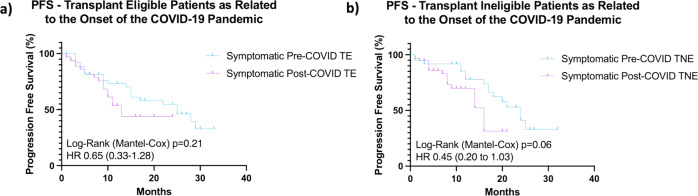
Kaplan-Meier estimates of progression-free survival (PFS) in patients with symptomatic MM as related to the onset of the COVID-19 pandemic. **a** Kaplan-Meier estimate of progression-free survival (PFS) in patients with symptomatic MM deemed fit for therapy and transplant eligible (*n* = 128) pre- and post-pandemic. Patients diagnosed 1st January 2019–31st January 2020 (Pre-COVID, *n* = 45) and from 1st February 2020–31st July 2021 (Post-COVID, *n* = 83). **b** Kaplan-Meier estimate of progression-free survival (PFS) in patients with symptomatic MM deemed fit for therapy, but transplant ineligible(*n* = 118). Patients diagnosed 1st January 2019–31st January 2020 (Pre-COVID, *n* = 41) and from 1st February 2020–31st July 2021 (Post-COVID, *n* = 77). PFS progression-free survival, TE transplant eligible, TNE transplant non-eligible.

Patients who were diagnosed with symptomatic myeloma pre-COVID were more likely to be treated with a triplet rather than doublet combination than those diagnosed in the post-COVID period (triplet pre-COVID 79.1%, post-COVID 63.75%; *p* = 0.014) (Fig. 5). The use of induction with oral lenalidomide with steroid increased in the post-COVID cohort (pre-COVID Len-Dex 17 (19.8%), post-COVID Len-Dex 53 (33.1%), *p* = 0.027), particularly in the early months of the pandemic (Supplementary Fig. 2). Rates of ASCT were lower in the post-COVID period (pre-COVID 22(25.6%) compared with post-COVID 22(13.8%), *p* = 0.024; Fig. 5). No significant difference in ORR between the two cohorts was seen for either transplant eligible or transplant-ineligible patients. Progression free survival (PFS) was not statistically different between pre-COVID and post-COVID patients, in either transplant eligible or ineligible groups. However, there was a non-significant trend to inferior PFS in the non-transplant-eligible patients diagnosed after the onset of the pandemic (HR 0.45(0.20–1.03), *p* = 0.06; Fig. 6), which may evolve with extended follow-up of this population.

**Fig. 7:**
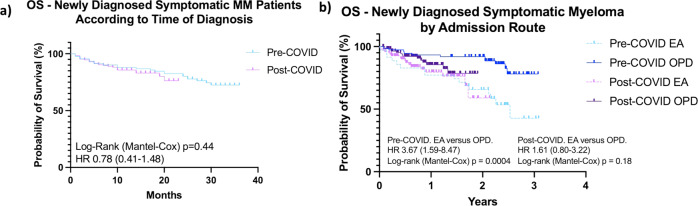
Kaplan-Meier estimates of overall survival (OS) of patients with symptomatic MM. Patient diagnosed from 1st January 2019–31st January 2020 (Pre-COVID, *n* = 93) and from 1st February 2020–31st July 2021 (Post-COVID, *n* = 172). **a** Overall survival of newly diagnosed symptomatic myeloma patients according to timing of diagnosis. **b** Overall survival of newly diagnosed symptomatic myeloma patients according to admission route. OPD diagnosed through outpatient route, EA Diagnosed through an emergency route.

**Fig. 8:**
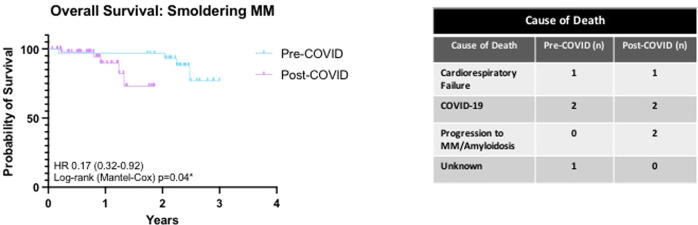
Overall survival (OS) of patients with smoldering MM. Kaplan Meier estimate of overall survival of smouldering myeloma patients diagnosed from 1st January 2019–31st January 2020 (Pre-COVID, *n* = 31) and from 1st February 2020–31st July 2021 (Post-COVID, *n* = 49). Table - Cause of death of smouldering myeloma patients according to timing of diagnosis.

For patients with symptomatic MM, deaths occurred in 25 (26.9%) patients in the pre-COVID cohort compared with 29/172 (16.9%) patients in the post-COVID cohort (HR 0.68 (0.41–1.1, Log-rank (Mantel–Cox *p* = 0.09, Fig. 7). This is likely to have been influenced by the duration of follow-up in the post-COVID cohort and requires ongoing monitoring. There were higher rates of death from cardio-respiratory failure in the pre-COVID cohort (pre-COVID 6 (22.2%), post-COVID 1 (3.13%); *p* = 0.04), however numbers were small. While no other significant difference in cause of death was identified between the two groups, the proportion of deaths due to progressive disease was higher in the post-COVID cohort, which warrants continued observation (Table 2). Overall survival (OS) at 1 year was not significantly different between the pre-COVID and post-COVID cohorts (Pre-COVID 88.2% compared with Post-COVID 87.8%, Fig. 7). In line with previously published data, OS was worse in patients who presented via an emergency route (Log-rank (Mantel–Cox) *p* = 0.0004), but no difference between the cohorts was evident (Fig. 7). Similarly, OS was impacted by the presence of frailty (Log-rank (Mantel–Cox) *p* = 0.005) with no difference between the cohorts noted (Supplementary Fig. 3). For patients with smoldering MM, deaths occurred in 3/30 (10%) of the Pre-COVID cohort and 7/50 (14%) of the post-COVID cohort (HR 0.17 (0.32–0.92) LogRank (Mantel–Cox), *p* = 0.04; Fig. 8). Infection with SARS-CoV2 accounted for the majority of deaths in smoldering MM patients.

**Table 2:** Cause of death for patients diagnosed from 01 January 2019–31 January 2020 (Pre-COVID, *n* = 110) and those diagnosed from 01 February 2020–31 July 2021 (Post-COVID, *n* = 213).

	Pre-COVID	Post-COVID
Mortality: *N* (%)	27 (24.5)	32 (15.0)
Progressive disease: *N* (%)	9 (33.3)	17 (53.1)
Infection:		
Sepsis	3 (11.1)	2 (6.2)
COVID-19	5 (18.5)	7 (21.9)
Cardio-respiratory failure	6 (22.2)	1 (3.1)
Cerebrovascular accident	0 (0.0)	1 (3.1)
Second primary malignancy	1 (3.7)	0 (0.0)
Upper GI bleed	0 (0.0)	1 (3.1)
Unknown	3 (11.1)	3 (0.9)

Where data were available regarding SARS-CoV2 infection (*n* = 284), 41 patients (14.4%) presented with symptoms and tested swab-positive (PCR) for symptomatic COVID-19, of which 12 patients (29.3%) died as a direct consequence of SARS-CoV2 infection. Infection was contracted a median of 6.8 months from diagnosis, with 17.1% contracting COVID-19 pre-diagnosis. COVID-19 infection occurred a median of 5.9 months after the 1st COVID-19 vaccination, with 22% of infections occurring pre-vaccination. The median time between the 1st and 2nd vaccination was 11 weeks (range 2.7–31.9 weeks). Vaccine response is beyond the scope of this audit.

## Discussion

Myeloma patients have been especially adversely impacted by the coronavirus pandemic. Already acquainted with the importance of infection control, they have been subject to prolonged shielding and often social isolation. Equally importantly, interactions with healthcare services, particularly primary care, have been altered or reduced. Accordingly, the likely impact of the pandemic on the health service delivery plan is a lengthening of diagnostic intervals and resultant delays in initiation of anti-myeloma therapy [19, 20]. Early in the pandemic, conservative estimates predicted that the pandemic itself, alongside social measures to limit its spread, would increase diagnostic delays in oncology and increase mortality rates [9, 21].

To date, the impact of the pandemic on diagnostic delay, clinical presentation, disease burden and disease-related morbidity has not been investigated in multiple myeloma (MM). We present the first data that examine, in the real-world setting, the likelihood of delays in diagnostic pathways and the impact on disease presentation alongside outcomes.

Our data demonstrate that patients diagnosed with myeloma during the COVID pandemic were more likely to present via the emergency route and with features of advanced disease, evidenced by increased frequency of lytic lesions and extramedullary disease. No difference was seen in the proportion of patients presenting with genetically high risk disease, though the level of missing data in the real-world setting is not insignificant in this dataset, despite the improved testing in the post-COVID cohort. In keeping with this, skeletal symptoms were the primary reason for presentation via the emergency route. Skeletal disease is associated with a significant morbidity burden including increased use of opiate-based pain relief, impaired mobility and a decrease in quality of life scores, while cancer-related pain has been reported to be associated with a triad of sleep disturbance, mood disturbance and fatigue [22–24]. Skeletal disease also has implications for healthcare-related costs, with one retrospective review reporting that skeletal event-related health costs made up 17% of a patient’s total treatment costs [25]. As a consequence, the increased rates of skeletal disease in the post-COVID patient cohort are anticipated to result in increased disease-related morbidity, impaired quality of life and increased healthcare-related costs.

After the UK index case of COVID-19, there were significantly more patients diagnosed with myeloma through an emergency route compared to elective, outpatient pathways. We have previously demonstrated the negative impact that this has on survivorship, resulting from a higher disease burden and reduce efficacy of therapies [4]. In this real-world, multi-region dataset, the greatest impact on mortality was seen within the first 6 months of diagnosis, where 25% had died compared to 10% of electively diagnosed MM patients. Whilst the current dataset corroborates the impact of route of diagnosis on survivorship, the time taken to reach diagnosis in relation to the onset of the pandemic was unchanged. This may in part be related to the limited follow-up, especially in the post-COVID cohort, the use of more modern anti-MM regimens (compared to regimens used as standard of care pre-2017 as reported [26]) and the confounding variable of non-MM related death, especially COVID-19, which has had devasting effects in the MM patient community [27, 28].

Patients presenting during the COVID pandemic were more frail than those presenting before the COVID pandemic. This is of particular relevance in MM where frailty and performance status are known to be associated with impaired treatment outcomes. Pawlyn et al. reported that performance status predicts treatment outcomes across all ages, highlighting the importance of biological frailty rather than chronological age [29], while the IMWG and UKMRA MRP frailty scores also demonstrate impaired survival in the more frail patient population [15, 16]. We, therefore, hypothesized that the post-COVID cohort of patients may experience impaired survival as a consequence of their increased frailty. When examined by IMWG frailty status, we demonstrated a significant impact on survivorship, but no temporal effect in relation to timing of diagnosis to date.

The impacts of COVID-19 pandemic on the diagnostic pathway and therapy delivery in MM were significant. Utilizing access to EPR from health care organizations, Martinez-Lopez and colleagues were able to highlight the incidence of newly diagnosed MM was reduced in 2020 compared to previous years and more importantly, survival of newly diagnosed MM decreased [30]. Interrogating multi-national datasets, the authors found that MM patients have been more severely impacted by COVID-19 pandemic than non-MM patients. In the UK, aiming to reduce hospital footfall, therapy delivery underwent a significant change, with an increase in doublet induction regimens, a preference for oral-based treatment strategies and a reduction in the rates of autografting [31], some of which may have been related to planned autograft deferrals. The current body of evidence serves to illustrate the impact of triplet, and more recently quadruplet induction regimens, followed by stem cell transplant consolidation to maximize depth and durability of response, as well as survivorship [32–34]. It is therefore anticipated that pandemic will leave a mark on myeloma survival in the years to come. Our data have started to uncover the impacts on response rates, but as yet show limited evidence of effect on durability of response or survivorship. There are limitations with the dataset included in this analysis, and the impact missing data has on the overall analysis and conclusions needs to be considered, though this is a typical limitation of real-world studies in MM [35]. The absence of a survival difference between cohorts may be because of the limited follow-up, or the sample size reported in this dataset. The clinical reality is that the true legacy of the COVID-19 pandemic is yet to be fully appreciated.

While the long-term impact of these changes may not become apparent for many years, it is clear that the COVID pandemic and the wider impact on the delivery of medical care, resulted in a shift in the symptomatology, disease burden and routes of diagnosis of patients presenting with myeloma and that this may have significant consequences for their long-term outlook. The full legacy of the SARS-CoV2 pandemic is still unfolding and has resulted in unprecedented strain on healthcare services. In seeking to address wider healthcare issues it is critical that rapid routes to diagnosis are established, and evidence-based, effective therapies made available to cancer patients. In this regard, the data presented here are important for physicians and policy makers alike.

## Supplementary information

aj-checklist

Supplementary Figure 1.

Supplementary Figure 2.

Supplementary Figure 3.
