# Supplementary material for: Delayed diagnosis resulting in increased disease burden in multiple myeloma: the legacy of the COVID-19 pandemic
Source: Blood Cancer J. 2023 Mar 15;13(1):38. doi: 10.1038/s41408-023-00795-w (PMC10015143; doi:10.1038/s41408-023-00795-w)
Supplement: Supplementary file 2 — Supplementary Figure 1. [file 41408_2023_795_MOESM2_ESM.pdf]

Supplementary Figure 1.

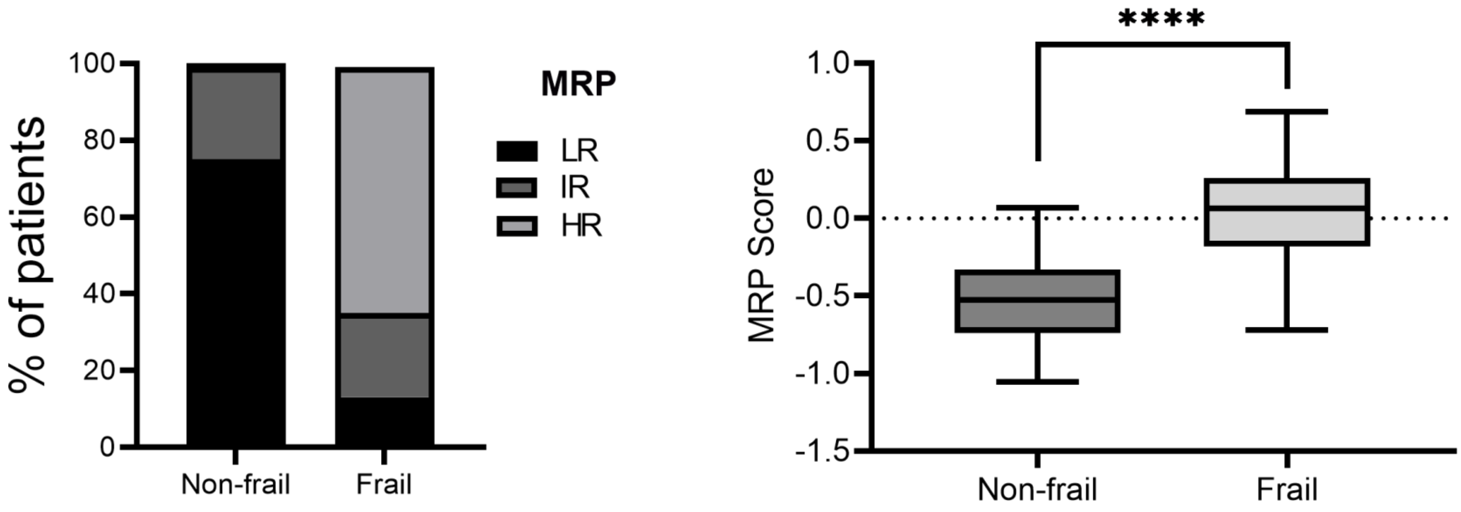

Supplementary Figure 1. UKMRA MRP values and concordance with mIMWG score. **HR** high risk, **IR** intermediate risk, **LR** low risk. ****p<0.0001.
