# Supplementary material for: Delayed diagnosis resulting in increased disease burden in multiple myeloma: the legacy of the COVID-19 pandemic
Source: Blood Cancer J. 2023 Mar 15;13(1):38. doi: 10.1038/s41408-023-00795-w (PMC10015143; doi:10.1038/s41408-023-00795-w)
Supplement: Supplementary file 4 — Supplementary Figure 3. [file 41408_2023_795_MOESM4_ESM.pdf]

Supplementary Figure 3

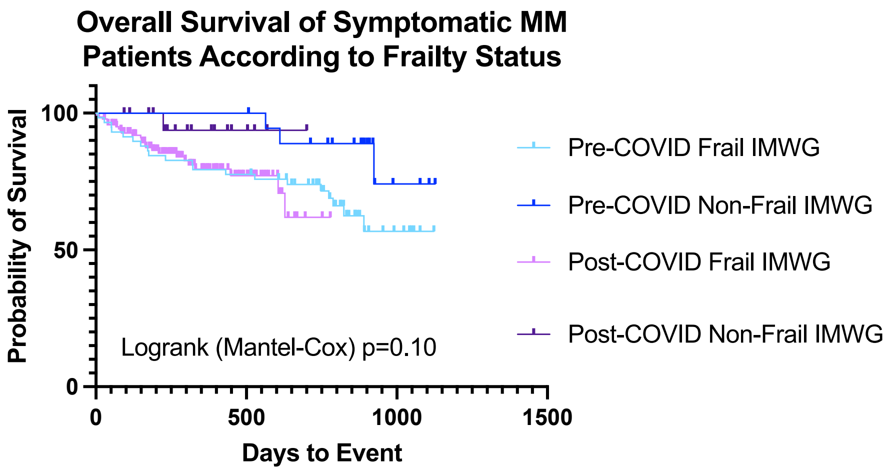

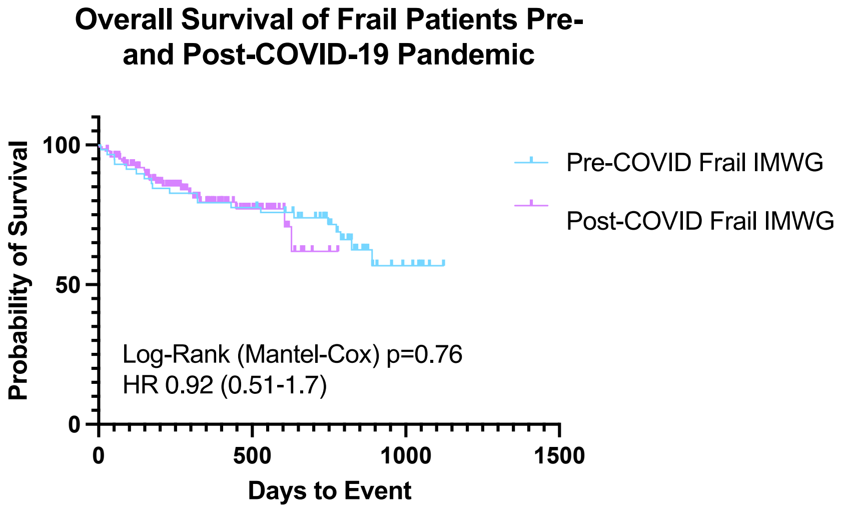

Supplementary Figure 3. Overall Survival of patients with symptomatic MM according to frailty, diagnosed from 1st January 2019 – 31st January 2020 (Pre-COVID) and from 1st February 2020 – 31st July 2021 (Post-COVID).
